# Participation of the Cholinergic System in the Development of Polycystic Ovary Syndrome

**DOI:** 10.3390/molecules26185506

**Published:** 2021-09-10

**Authors:** Rosa Linares, Xóchitl N. Acuña, Gabriela Rosas, Elizabeth Vieyra, Deyra A. Ramírez, Andrea Chaparro, Julieta A. Espinoza, Roberto Domínguez, Leticia Morales-Ledesma

**Affiliations:** 1 Physiology of Reproduction Laboratory, Biology of Reproduction Research Unit, Facultad de Estudios Superiores Zaragoza, UNAM, AP 9-020, Mexico City 15000, Mexico; rosa_linaresc@yahoo.com.mx (R.L.); andmara_24@hotmail.com (X.N.A.); gabriela_rosasg@yahoo.com.mx (G.R.); eliz_vv@hotmail.com (E.V.); ych022003@yahoo.com.mx (A.C.); azuespinozamoreno@hotmail.com (J.A.E.); 2 Laboratorio de Endocrinologia, Biology of Reproduction Research Unit, Facultad de Estudios Superiores Zaragoza, UNAM, AP 9-020, Mexico City 15000, Mexico; 3 Laboratorio de Investigación en Cronobiología y Reproducción, Biology of Reproduction Research Unit, Facultad de Estudios Superiores Zaragoza, UNAM, AP 9-020, Mexico City 15000, Mexico; rdcasala@hotmail.com; 4 Facultad de Estudios Superiores Zaragoza Campus III, UNAM, San Miguel Contla 90640, Mexico; deyra_veg@yahoo.com.mx

**Keywords:** PCOS, acetylcholine, atropine, ovulation, steroidogenesis

## Abstract

In rats with polycystic ovary syndrome (PCOS) induced by injection of estradiol valerate (EV), unilateral or bilateral section of the vagus nerve restores ovulatory function in 75% of animals, suggesting that the vagus nerve participates in the development of PCOS. Since the vagus nerve is a mixed nerve through which mainly cholinergic-type information passes, the objective of the present study was to analyze whether acetylcholine (ACh) is involved in the development of PCOS. Ten-day-old rats were injected with 2.0 mg EV, and at 60 days of age, they were microinjected on the day of diestrus in the bursa of the left or right ovary with 100 or 700 mg/kg of ovarian weight atropine, a blocker of muscarinic receptors, and sacrificed for histopathological examination after the surgery. Animals with PCOS microinjected with 100 mg of atropine showed a lack of ovulation, lower serum concentrations of progesterone and testosterone, and cysts. Histology of the ovaries of animals microinjected with 700 mg of atropine showed corpus luteum and follicles at different stages of development, which was accompanied by a lower concentration of progesterone and testosterone. These results allow us to suggest that in animals with PCOS, ACh, which passes through parasympathetic innervation, is an important component in the persistence and development of the pathophysiology.

## 1. Background

Polycystic ovary syndrome (PCOS) is an endocrinopathy that, according to the Rotterdam consensus, has an incidence of 15 to 18%. Based on the Rotterdam consensus, in women, the diagnosis of PCOS requires the presence of at least two of these criteria: the presence of clinical or biochemical hyperandrogenism, ovulatory dysfunction, and polycystic ovarian morphology [1,2]. In the diagnosis of PCOS, some exclusion criteria for hyperandrogenism should be considered, including nonclassic adrenal hyperplasia, idiopathic hirsutism, and premature adrenarche [3]. An experimental model proposed to study PCOS is the administration of estradiol valerate (EV) to infantile or adult rats. EV is a long-acting estrogen. Injecting 2 mg of EV into infantile or adult rats results in the interruption of the estrus cycle, persistent vaginal cornification, anovulation, the formation of follicular cysts, and high concentrations of testosterone [4,5,6].

The etiology of PCOS in humans is unknown; however, various mechanisms involved in the alterations that accompany the development of the pathophysiology have been described. The most accepted hypotheses about its etiology are modifications of the secretion pulses of gonadotropin-releasing hormone (GnRH), alteration of the secretion of insulin or its effects on the secretion of androgens by the ovaries, and the hyperactivity of the sympathetic fibers that innervate the gonads [7,8].

The luteinizing hormone (LH) peak on proestrus afternoon (12:00–17:00 h) is the result of an increase in the frequency and amplitude of the GnRH secretion pulses in the preoptic-hypothalamic-anterior area (POA-AHA) in response to several molecules that influence the neural network of GnRH [9], such as acetylcholine (ACh).

ACh is synthesized in the ovaries [10,11] and is also supplied by extrinsic innervation [12]. The main contribution of ACh to the ovary comes from the vagus nerve. Currently, the ovarian structures that receive vagal innervation are unknown; however, it has been shown in prepubertal or adult rats that nervous information that reaches the ovary via the vagus nerve participates in the regulation of ovulation and the secretion of steroid hormones [13,14,15]. Apparently, ACh is involved in the development and persistence of the syndrome, since unilateral or bilateral section of the vagus nerve results in the reestablishment of ovulation, as demonstrated by the presence of a corpora lutea [16,17].

ACh acts on nicotinic and muscarinic receptors. In the ovaries of women and rats, muscarinic receptors were found to correspond to subtypes M1, M3, and M5. The M3 receptor is located in oocytes, while M1 and M5 are found in granulosa cells [18].

Atropine is a nonselective antagonist of muscarinic receptors and it inhibits the effects of ACh by blocking its binding with muscarinic receptors found in effector cells, parasympathetic neuroeffector junctions, peripheral ganglia, and the central nervous system (CNS). Atropine does not bind to nicotinic receptors [19].

In adult rats in diestrus 1 or diestrus 2, a dose of 100 mg/kg atropine injected subcutaneously blocks ovulation in 100% of animals, whereas a dose of 700 mg/kg is required on the day of proestrus to block ovulation in more than 80% of animals, suggesting a relationship between gonadotropin secretion during the estrus cycle and the cholinergic system [20].

In the present study, the role of the cholinergic system in ovulation and steroidogenesis was analyzed in rats with PCOS induced by the administration of EV through blocking the muscarinic receptors of the ovary by intrabursal microinjection of 100 or 700 mg of atropine.

## 2. Materials and Methods

Female rats of the CII-ZV strain were kept under controlled lighting conditions (14 light hours for 10 h of darkness, lights on from 05:00 h to 19:00 h) at a temperature of 22 °C. During the experiment, they were treated with the applicable regulations for the use of experimental animals (NOM-062-ZOO-1999, Technical specifications for the production, care, and use of laboratory animals). The experimental protocols used in this study were approved by the Bioethics Committee of the Facultad de Estudios Superiores-Zaragoza, Universidad Nacional Autónoma de México. All possible efforts were made to minimize the number of animals used and their suffering.

Neonate animals were placed in litters of five females and one male. Females at 10 days of age were injected intraperitoneally with a 2.0 mg dose of EV (Sigma Chemical Co., St. Louis, MO, USA) dissolved in 0.1 mL of sesame oil. The offspring had free access to the mother until 24 days of age (day of weaning) and subsequently had free access to food and water until the day they were autopsied. One day after EV administration, monitoring of the vaginal opening began. The injection of EV causes an advance in the age of the vaginal opening [21]; therefore, once canalization of the vagina occurs, daily vaginal smears are taken for a period of 8 days to verify the acyclicity of the animals caused by the injection of EV. The smears were retaken 2 weeks before the animals were 60 days old. Given the acyclicity of these animals, the criterion for atropine microinjection into the left or right ovarian bursa was the presence of diestrus preceded by proestrus.

To do this, following the previously described methodology [22], each of the rats underwent a unilateral (left or right) laparotomy under general anesthesia, and the ovaries were exteriorized to enable microinjection of 20 μL of 100 or 700 mg/kg ovarian weight (ow) atropine (Sigma Chemical Co., St. Louis, MO, USA) dissolved in saline, which served as the vehicle. The microinjection was made with the aid of a Nano-Injector, Stepper Motorized (CMA/100; BAS, Estocolmo, Suecia) and a 100 μL microsyringe (Hamilton, Reno, NV, USA) equipped with a 25-gauge needle; the injection rate was 4 μL/min. To prevent fluid leakage, the needle was kept in the ovarian bursa for 2 min. Subsequently, the ovaries were carefully cleaned, dried, and returned to the abdominal cavity, and the skin and muscle were sutured. The surgeries were performed between 9:00 and 11:00 a.m. The number of animals used in each experimental group was as follows: EV, *n* = 10; EV + ATR 100 mg in left ovarian bursa (LB), *n* = 10; EV + ATR 100 mg in right ovarian bursa (RB), *n* = 6; EV + ATR 700 mg LB, *n* = 9; and EV + ATR 700 mg RB, *n* = 9.

### 2.1. Autopsy Procedure

After surgery, all animals were sacrificed at 61–64 days of age after a vaginal smear indicated estrus. All rats in the study were sacrificed by decapitation. Blood from the trunk was collected and allowed to clot at room temperature for 30 min and then centrifuged at 3500 rpm for 15 min. The serum obtained was frozen at −20 °C until quantification of progesterone and testosterone. At the time of the autopsy, the ovaries were dissected and weighed, the oviducts were dissected, and the number of oocytes ovulated was counted with the aid of a dissecting microscope (Nikon, model SMZ800, Tokyo, Japan), following the usual laboratory methodology [23].

### 2.2. Morphometric Analysis of the Ovaries

The left and right ovaries of all rats used in the study were removed, cleaned of adherent fat tissue, weighed (accuracy of 0.001 mg), and subsequently immersed in Bouin’s fixative solution for 24 h, after which they were sequentially placed in 70%, 96%, and 100% ethanol and chloroform. The tissues were then embedded in paraffin wax. The left and right ovaries of three randomly selected rats from each group were serially sectioned at 10 μm thick, mounted, and stained with hematoxylin-eosin, and morphometric analysis was performed with the aid of a binocular microscope (Nikon, Model Labophot-2). Follicles were considered atretic when demonstrating at least one of the following characteristics: nuclear pyknosis of the granulosa cells, desquamation of the granulosa cells in the antral cavity, or hyperplasia of the thecal cell layers [24]. Those follicles that had a broad antral cavity, a decrease in granulosa cell layers, and an enlarged thecal cell layer, as well as an absence of an oocyte, were considered cysts. Precystic follicles have a large antral cavity with or without an oocyte, four or five layers of granulosa cells, normal-looking theca, and invaginations and evaginations of the follicular wall [25]. All micrographs were taken with a digital camera (Nikon, DS-U2, Japan).

### 2.3. Hormone Quantification

Following the protocol provided by the manufacturer, conventional ELISA procedures and commercial kits (Enzo Life Sciences Inc., Farmingdale, NY, USA) were used to measure the serum progesterone and testosterone levels. The intra- and interassay coefficients of variation were 7.52 and 8.41% for progesterone and 6.42 and 7.32% for testosterone, respectively.

### 2.4. Statistical Analysis

Statistical analyses were performed using GraphPad InStat 3 Software, Inc. (San Die-go, CA, USA). The percentage of ovulating animals was analyzed using Fisher’s exact probability test. Data on the number of oocytes released and the number of total, healthy, atresic, precystic and cystic follicles were analyzed with a Kruskal–Wallis test followed by Dunn’s test. The steroid hormone levels in the serum were analyzed using one-way analysis of variance (ANOVA) followed by Tukey’s test. When two means were compared, we used Student’s *t*-test or a Mann–Whitney U test. Values of *p* ≤ 0.05 were considered statistically significant. Data are expressed as the mean ± standard deviation (S.D.).

## 3. Results

We have previously shown that EV administration results in alteration of the estrus cycle, ovulation blockage, hyperandrogenism, and the presence of ovarian cysts [16,21].

### 3.1. Estrus Cycle

As we have already shown before [16,21], injection with EV alters the estrus cycle pattern with a predominance of days in diestrus or estrus. Microinjection of 100 or 700 mg/kg/ow atropine did not reverse the effect of EV, since less than 20% of treated animals had cycles 4 days after surgery.

### 3.2. Ovulatory Response

**Figure 1:**
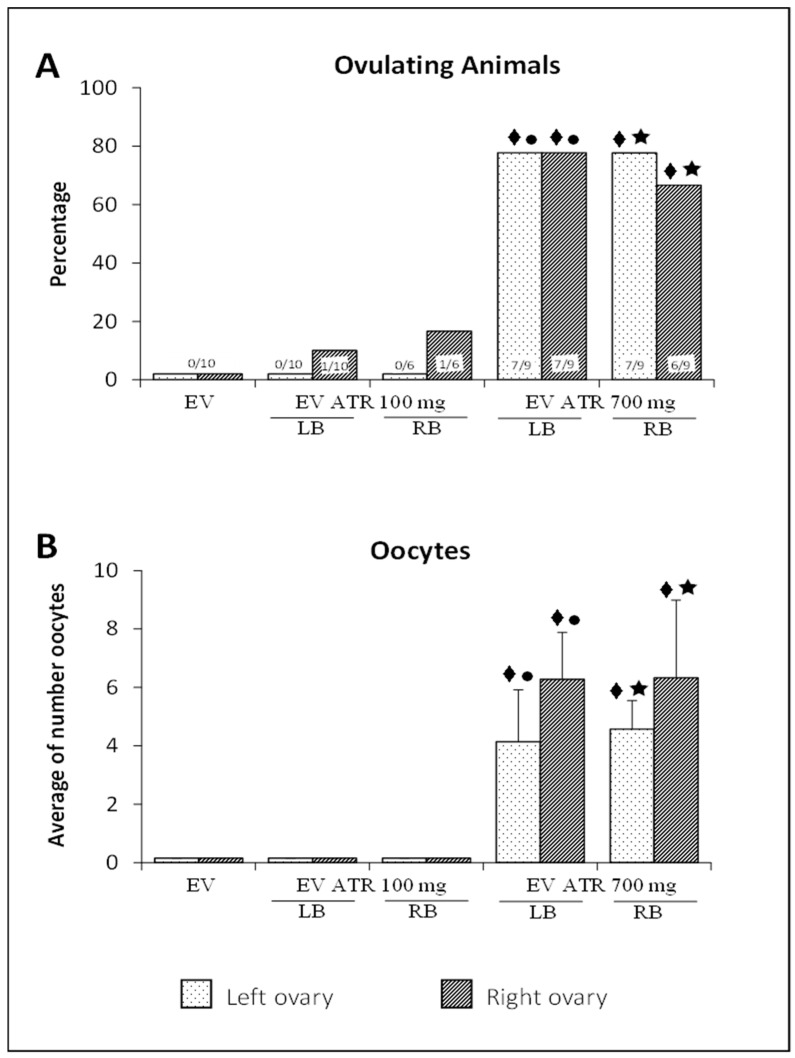
Percentage of ovulating animals (**A**) and mean ± S.D. of the number of oocytes released (**B**) by the left or right ovary of rats treated with estradiol valerate (EV) at 10 days of age, subjected to the microinjection of 100 or 700 mg/kg/ow atropine (ATR) in the left (LB) or right (RB) ovarian bursa at 60 days of age (on the diestrus day), and sacrificed at 61–64 days of age after a vaginal smear indicated estrus. The numbers at the base of the bars indicate the number of ovulating animals/number of treated animals. ⧫ *p* < 0.05 vs. EV (their respective ovary); ● *p* < 0.05 vs. EV ATR 100 mg LB (their respective ovary); ★ *p* < 0.05 vs. EV ATR 100 mg RB (their ovary respective). Fisher’s test for ovulation percentage; Kruskal–Wallis followed by Dunn’s test for number of oocytes. EV, *n* = 10; EV + ATR 100_LB, *n* = 10; EV + ATR 100_RB, *n* = 6; EV + ATR 700_LB, *n* = 9; EV + ATR 700_RB, *n* = 9.

Ovulation was blocked in 100% of the animals injected with EV. Only 10% of the animals treated with EV and microinjected with 100 mg of atropine ovulated; when the microinjection was made in the left ovarian bursa, one animal ovulated five oocytes in the right ovary, and when they were microinjected in the bursa of the right ovary, one animal ovulated two oocytes in the right ovary. On average, more than 70% of the animals ovulated in both ovaries when they were microinjected with 700 mg of atropine (Figure 1A). In a cyclic adult animal, the left ovary releases an average of six oocytes, and the right ovary releases an average of four oocytes [26]. This rate is similar to that observed in animals injected with EV and microinjected with 700 mg of atropine in the left or right ovarian bursa (Figure 1B).

### 3.3. Steroid Hormone Concentration

#### 3.3.1. Progesterone

**Figure 2:**
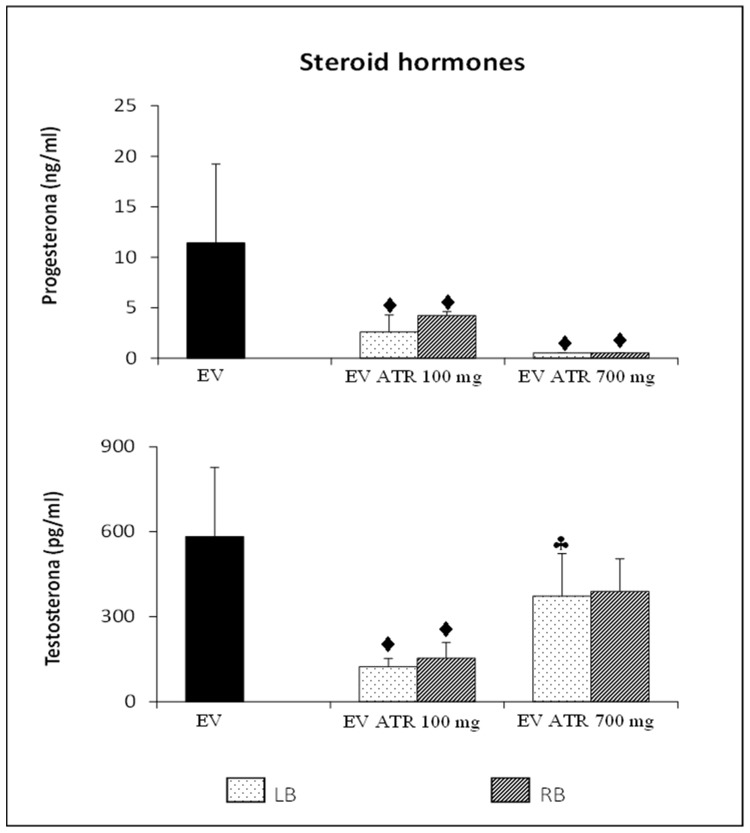
Mean ± S.D. of the concentration of progesterone (ng/mL) and testosterone (pg/mL) of rats treated with estradiol valerate (EV) at 10 days of age, subjected to the microinjection of 100 or 700 mg/kg/ow of atropine (ATR) in the left (LB) or right (RB) ovarian bursa at 60 days of age (on the diestrus day), and sacrificed at 61–64 days of age after a vaginal smear indicated estrus. ⧫ *p* < 0.05 vs. EV; ♣ *p* < 0.05 vs. EV + ATR 100 mg (LB), one-way ANOVA followed by Tukey’s multiple comparison. EV, *n* = 10; EV + ATR 100_LB, *n* = 10; EV + ATR 100_RB, *n* = 6; EV + ATR 700_LB, *n* = 9; EV + ATR 700_RB, *n* = 9.

Microinjection of 100 or 700 mg/kg/ow atropine into the left or right ovarian bursa of animals previously treated with EV resulted in a lower concentration of progesterone than animals injected with EV alone. This effect was more pronounced in the microinjected animals at 700 mg/kg/ow (Figure 2).

#### 3.3.2. Testosterone

Blocking muscarinic receptors with 100 mg/kg/ow atropine in the ovaries of rats treated with EV resulted in a decrease in the hyperandrogenic condition observed in animals injected only with EV. This effect was not observed in animals microinjected with 700 mg/kg/ow atropine, since their testosterone concentration was similar to that of animals injected only with EV. Microinjection with 700 mg of atropine in the left bursa resulted in the highest concentration of the hormone compared to the animals microinjected with 100 mg of atropine (Figure 2).

### 3.4. Follicular Population Analysis

**Figure 3:**
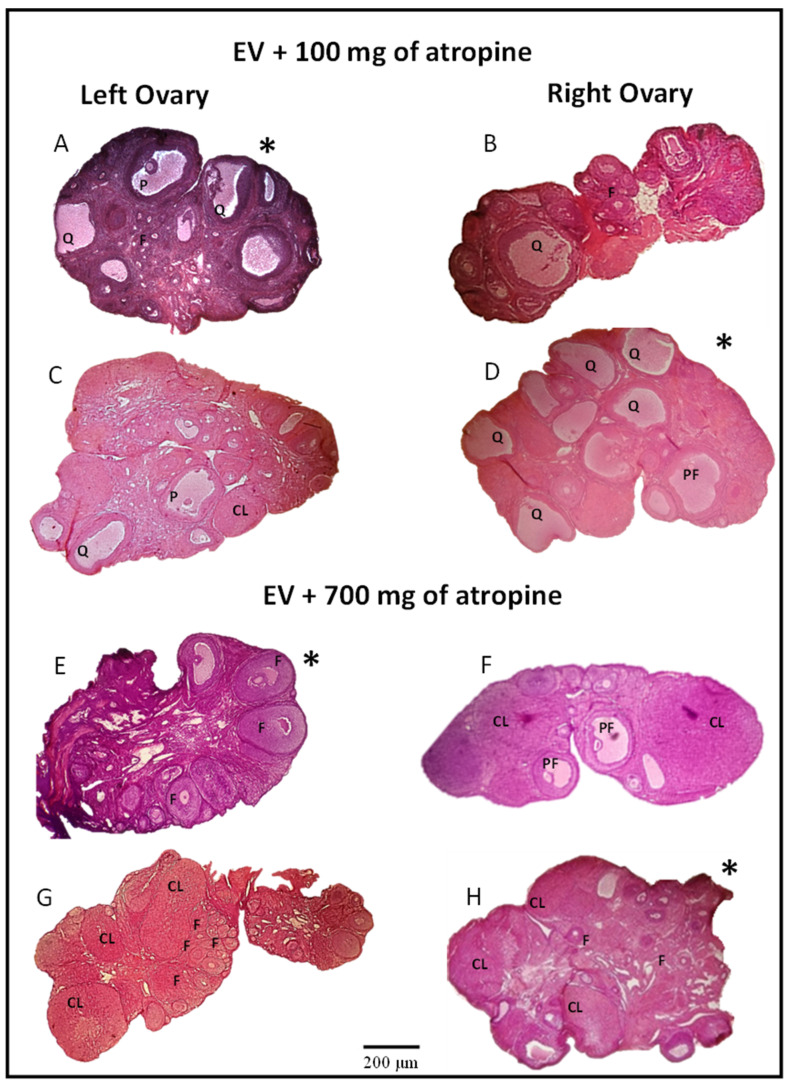
Photomicrographs at 40× magnification from the ovaries of rats treated with estradiol valerate (EV) at 10 days of age and subjected to the microinjection of 100 mg/kg/ow atropine in the left ovarian bursa (**A**) or right ovarian bursa (**D**) or with 700 mg/kg/ow atropine in the left ovarian bursa (**E**) or right ovarian bursa (**H**) at 60 days of age (on the day of diestrus). Their respective contralateral ovaries (**B**,**C**,**F**,**G**) are also shown. All animals were sacrificed at 61–64 days of age after a vaginal smear indicated estrus. CL: corpus luteum; PF: preovulatory follicle; F: developing follicles; Q: cyst; P: precystic. 4× microscopic lens. Scale bar = 200 µm. The (*) indicates the microinjected ovary with ATR. EV, *n* = 10; EV + ATR 100_LB, *n* = 10; EV + ATR 100_RB, *n* = 6; EV + ATR 700_LB, *n* = 9; EV + ATR 700_RB, *n* = 9.

Previously, it has been reported that EV injection results in the development of ovarian cysts and the absence of a corpus luteum [16,21,27]. Our results show that the microinjection of 100 mg of atropine into the left or right ovary results in the development of follicles in the microinjected ovary (Figure 3A,D), while cystic structures and corpora lutea were observed in the contralateral ovary (Figure 3B,C).

Blocking muscarinic receptors by the microinjection of 700 mg of atropine into the bursa of the left or right ovary of animals previously treated with EV reduces cystic structures and reactivates follicular growth in the microinjected ovary (Figure 3E,H) and in the ovary contralateral to microinjection (Figure 3F,G).

**Figure 4:**
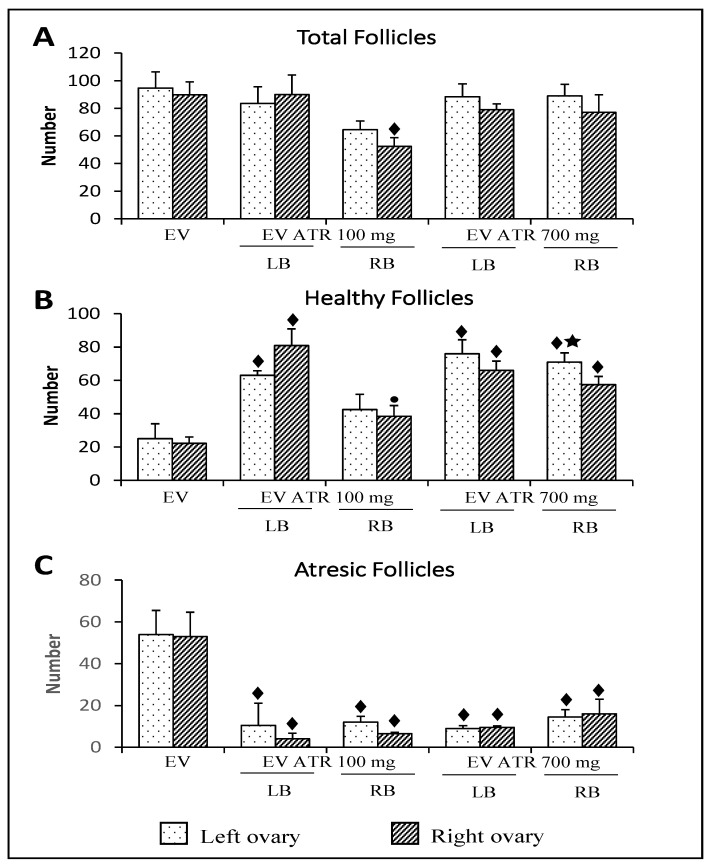
Mean ± S.D. of the number of total (**A**), healthy (**B**), and atresic (**C**) follicles measured in the ovaries of rats treated with estradiol valerate (EV) at 10 days of age, subjected to the microinjection of 100 or 700 mg/kg/ow of atropine (ATR) in the left (LB) or right (RB) ovarian bursa at 60 days of age (on the diestrus day), and sacrificed at 61–64 days of age after a vaginal smear indicated estrus (*n* = 3 animals per group). ⧫ *p* < 0.05 vs. EV (their ovary respective); ● *p* < 0.05 vs. EV ATR 100 mg LB (their ovary respective); ★ *p* < 0.05 vs. EV ATR 100 mg RB (their ovary respective), Kruskal–Wallis test followed by Dunn’s test.

The total number of follicles in the right ovary of animals previously treated with EV and microinjected in the right ovarian bursa with 100 mg of atropine was lower than that in animals injected only with EV (Figure 4A).

In comparison with the animals injected only with EV, the microinjection of 100 mg of atropine in the left bursa or 700 mg of atropine in the left or right bursa resulted in more healthy follicles. In animals microinjected with 100 mg of atropine in the right bursa, the number of healthy follicles was lower than that in the animals injected in the left bursa (Figure 4B).

The microinjection of 100 mg or 700 mg of atropine resulted in a lower number of atresic follicles compared to animals injected with EV (Figure 4C).

**Figure 5:**
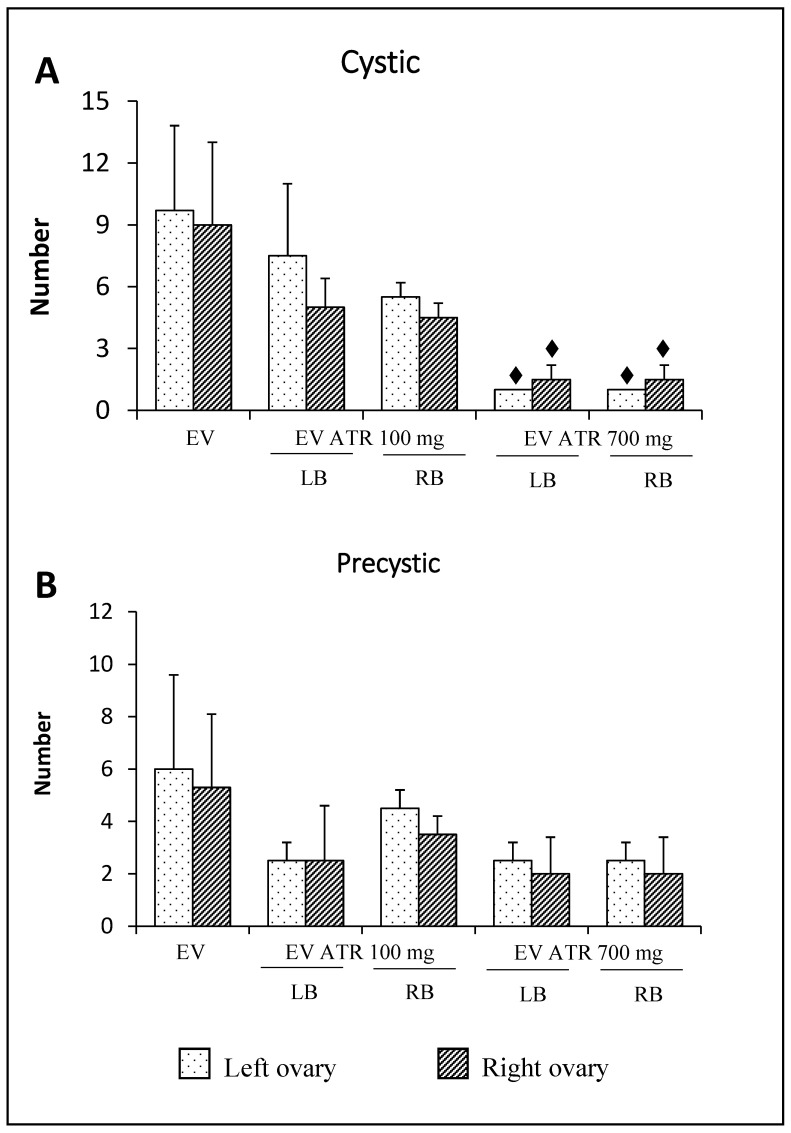
Mean ± S.D. of the number of cystic (**A**) and precystic (**B**) structures measured in the ovaries of rats treated with estradiol valerate (EV) at 10 days of age, subjected to the microinjection of 100 or 700 mg/kg/ow of atropine (ATR) in the left (LB) or right (RB) ovarian bursa at 60 days of age (on the diestrus day), and sacrificed at 61–64 days of age after a vaginal smear indicated estrus (*n* = 3 animals per group). ⧫ *p* < 0.05 vs. EV (their ovary respective), Kruskal–Wallis test followed by Dunn’s test.

The number of cysts in animals microinjected with 700 mg of atropine in the left or right bursa was lower than that in animals injected only with EV (Figure 5A). The number of precysts was not modified by atropine treatment (Figure 5B).

## 4. Discussion

The results of the present study show that in animals with PCOS induced by EV injection, the cholinergic system stimulates the development of hyperandrogenism, which characterizes the pathophysiology, while the role of the cholinergic system in ovulation depends on the available receptors.

In our experimental model, the administration of a dose of EV resulted in an increase in the serum concentration of testosterone, which was accompanied by a lack of ovulation and the development of ovarian cysts. The diagnostic characteristics of PCOS in women [1], as presented in the introduction, were induced in rats by EV injection [28].

We previously showed that in animals with PCOS pathophysiology, unilateral or bilateral vagotomy performed in 24-day-old rats—the effects of which were evaluated in the adult stage—induced ovulation in 75% of the animals and reversed the cystic condition. These results allowed us to suggest that the information carried by the vagus nerve to the ovaries participates in the regulation and maintenance of the syndrome [16]. Since ACh is the predominant neurotransmitter of the vagus nerve, it is possible that the maintenance of this syndrome may be the result of activation of the cholinergic system. Similarly, it has been reported that in cyclic rats, the vagus nerve participates in the regulation of steroidogenesis, follicular development, and ovulation [13,14,15,29,30].

Affinity is understood as the ability of a drug to bind to a specific receptor and form a drug–receptor complex. In studies of the cholinergic system, it has been observed that the effects of the various cholinergic drugs depend on the concentration used. The studies carried out by Stillman et al. [31] showed that the microinjection of methoctramine, a specific antagonist of M2 receptors, depends on the concentration used; a concentration of 16 µM resulted in a greater release of acetylcholine, while a concentration of 0.50 µM resulted in a lower release. These results led the authors to postulate that the effects of cholinergic antagonists on acetylcholine release depend on the concentration used [31]. This result is consistent with other in vivo investigations that showed increased acetylcholine levels after subcutaneous administration of nonselective cholinergic antagonists, such as scopolamine [32]. Similar increases in acetylcholine have also been reported in the rat striatum after intraperitoneal administration of atropine [33]. In the present study, it was shown that in rats with PCOS, blocking of muscarinic receptors by the microinjection of 100 mg of atropine in the bursa of the left or right ovary was not able to induce ovulation. However, when the atropine dose was increased to 700 mg, more than 70% of the animals ovulated in both ovaries. Therefore, it is possible that the observed response to ovulation is because the availability of the muscarinic receptors is different depending on the dose used.

We have previously shown that in an animal model of PCOS induced by the administration of EV, the concentration of progesterone is similar to that of an animal without pathology, although the ovarian histology of the animals injected with EV presents follicular cysts and an absence of the corpora lutea [16]. The progesterone concentration may not change because most progesterone comes from the adrenal glands rather than the ovaries [34]. In animals with PCOS microinjected with 100 mg atropine, the lack of ovulation was corroborated by the absence of a corpus luteum, which would explain the lower concentration of progesterone observed in these animals. The animals that received 700 mg of atropine ovulated, but their progesterone concentration was low. The studies by Burden and Lawrence [35] suggest that the vagus nerve regulates the activity of enzyme 3β-HSD (an enzyme that participates in the synthesis of progesterone) in a stimulating way, since bilateral section of the vagus nerve in pregnant rats decreased the 3β-HSD activity in the interstitial gland and corpus luteum, accompanied by a decrease in progesterone concentration. Given that the vagus nerve is a mixed nerve and most of its fibers are cholinergic in nature [36], our results allow us to suggest that in PCOS rats, ACh participates in a stimulating mechanism that regulates progesterone secretion.

In the steroidogenesis pathway, the formation of cAMP is essential for the activation of PKAs and the subsequent phosphorylation of the regulatory protein of acute steroidogenesis (StAR), an enzyme that intervenes in the transport of cholesterol to the inner membrane of the mitochondria. ACh participates in the regulation of the action of enzymes involved in steroidogenesis. Increased StAR activity by human chorionic gonadotropin (hCG) is amplified in the presence of carbachol, a cholinergic agonist, resulting in increased progesterone secretion [37]. Human and nonhuman primate ovarian granulosa cells display M1, M3, and M5 receptors. The oocyte has the M3 receptor bound to Gq proteins. The presence of receptors in both ovarian structures suggests that the ACh produced by luteinized or nonluteinized granulosa cells acts in a paracrine/autocrine manner on oocytes and granulosa cells [38]. The function of muscarinic M1/M5 receptors has been shown with Ca^2+^ measurements in human granulosa cells. The addition of agonists to carbachol produces an increase in intracellular Ca^2+^ concentrations, while the administration of pirenzepine (a selective antagonist of the M1 receptor) prevents the elevation of Ca^2+^ induced by ACh [39]. In luteinized granulosa cells and luteal cells of various species, there are Ca(^2+^)-activated K(^+^) channels (BKCa). Blockade of receptors with iberiotoxin (a BKCa channel blocker) decreases LH-dependent progesterone secretion. Activation of BKCa channels requires a cholinergic stimulus [40]. The proposed mechanism by which BKCa channels mediate steroidogenesis is the following: the ACh produced by granulosa cells after binding to the ovarian M1/M5 receptors stimulates the release of intracellular Ca^2+^, which results in the activation of the BKCa receptors, activation of voltage-gated channels, and hyperpolarization of the membrane, which translates into the secretion of progesterone and estradiol in the presence of LH [41]. In the present study, blocking the cholinergic system, either in the left or right ovarian bursa of animals with PCOS, reduced the serum concentration of testosterone compared to an animal injected only with EV. It is possible that such a decrease in the concentration of steroids could be related to the blocking of muscarinic receptors by atropine microinjection, which prevents ACh from activating the signaling cascade to increase Ca^2+^, thereby resulting in low steroidogenic activity being observed.

The functional capacity of the ovaries is asymmetric. The left ovary releases more oocytes than the right ovary [26], and their capacity to secrete steroid hormones varies depending on the day of the estrous cycle [13,34,42,43]. The main difference between the right and left ovaries is related to their ability to modulate the signals of the neuroendocrine system, which participate in the regulation of its functions. These regulatory differences are related to the innervations received by each ovary and their communication with the CNS [44]. In Chinese hamster ovary (CHO) cells stably transfected with M2 or M4 muscarinic receptors, the muscarinic agonist carbachol has inhibitory and stimulating effects on the synthesis of cyclic AMP. Although carbachol inhibits cyclic AMP synthesis at low concentrations, its effect diminishes at high concentrations (causing an upward inflection of the concentration–response curve) and becomes stimulating after cells are pretreated with pertussis toxin [45]. In the present study, we have shown that blocking the cholinergic system with 100 mg of atropine, either in the right or left ovarian bursa of animals with PCOS, reduces the serum concentration of testosterone, while in rats injected with 700 mg of atropine into the left ovarian bursa, the testosterone concentration was higher compared to those who received a 100 mg injection of atropine. These effects may be due to asymmetries of the ovaries in terms of nerve communication and the dose of atropine used.

Bódis et al. [46] showed that ACh treatment significantly increased progesterone and estradiol secretion and that these effects were blocked by atropine. A similar experimental design showed that ACh has a direct modulatory effect on gonadotropin-stimulated steroid production in granulosa cells [47]. Similar findings were reported in ruminants, where the continuous exposure of granulosa cells to ACh resulted in dose-dependent increases in the secretion of progesterone [48]. On the other hand, according to Cruz et al. [49], animals injected in the left ovary with pirenzepine did not show any changes in progesterone or estradiol serum concentrations 1 h after the treatment, suggesting that it is necessary to quantify the levels of both hormones at longer intervals after M1R blockade to rule out that ACh binding to M1 receptors regulates hormone secretion. Unlike what was obtained with 700 mg of atropine, where reactivation of follicular growth was observed, blockage of the muscarinic system by microinjection of 100 mg did not modify the histological appearance of the microinjected ovary compared to that of the animals treated with EV alone. This discrepancy may be due to the available muscarinic receptors being modified in response to the concentration of atropine used.

Even though atropine was injected into only one of the ovaries, the morphology of the contralateral gonad did not correspond to that of an animal with PCOS. Regardless of the dose of microinjected atropine, when the contralateral ovary was the right ovary, small follicles and precystic structures with signs of atresia were observed. A different ovarian morphology was observed when the contralateral ovary was the left ovary, where follicles are seen at different stages of development and sometimes even a corpus luteum. These results are further evidence of possible communication between the gonads, as was previously suggested by Morales [50].

Riquelme [51] showed that when ACh esterase was blocked in rats exposed to cold stress (4 °C for 3 h day for 28 days), ovarian levels of both NA and ACh were elevated, and fewer cysts and normal testosterone and estradiol plasma levels were found. In the present study, it was observed that the blockage of the cholinergic system by the administration of atropine to animals previously treated with EV increased the number of healthy follicles and decreased follicular atresia, as well as the number of cysts. These results suggest that ACh stimulation regulates the persistence of PCOS, since its blockade reverses the formation of cysts induced by estradiol-increased sympathetic activity, as has previously been shown [25]. Furthermore, it is clear that the role of ACh depends on the endocrine environment of the animal.

Taken together, the present results show that in adult animals with PCOS, the cholinergic system modulates the secretion of steroid hormones in a stimulating way, while its participation in the regulation of the mechanisms that lead to follicle rupture appears to depend on the availability of the muscarinic receptors. However, additional studies are necessary to elucidate whether the effect of ACh is related to a difference in the expression of muscarinic receptors in the ovaries or to their availability for ACh. For this, we propose continuing to study the role of the cholinergic system in the regulation of PCOS, both in vivo and in vitro.

## 5. Strengths and Limitations

The present study lays a foundation to analyze the possibility of using blockade of the cholinergic system at the ovarian level as a therapeutic treatment to induce ovulation in women with PCOS. Since we used a nonselective cholinergic blocker, atropine, we need to conduct additional studies to determine the type of muscarinic receptor involved in this mechanism.

## Data Availability

Not applicable.

## References

[1] Bremer A.A. (2010). Polycystic Ovary Syndrome in the Pediatric Population. Metab. Syndr. Relat. Disord..

[2] Goodarzi M.O., Dumesic D.A., Chazenbalk G., Azziz R. (2011). Polycystic Ovary Syndrome: Etiology, Pathogenesis and Diagnosis. Nat. Rev. Endocrinol..

[3] Azziz R., Carmina E., Dewailly D., Diamanti-Kandarakis E., Escobar-Morreale H.F., Futterweit W., Janssen O.E., Legro R.S., Norman R.J., Taylor A.E. (2009). The Androgen Excess and PCOS Society Criteria for the Polycystic Ovary Syndrome: The Complete Task Force Report. Fertil. Steril..

[4] Barria A., Leyton V., Ojeda S.R., Lara H.E. (1993). Ovarian Steroidal Response to Gonadotropins and Beta-Adrenergic Stimulation Is Enhanced in Polycystic Ovary Syndrome: Role of Sympathetic Innervation. Endocrinology.

[5] Rosa-e-Silva A., Guimaraes M.A., Padmanabhan V., Lara H.E. (2003). Prepubertal Administration of Estradiol Valerate Disrupts Cyclicity and Leads to Cystic Ovarian Morphology during Adult Life in the Rat: Role of Sympathetic Innervation. Endocrinology.

[6] Sotomayor-Zárate R., Dorfman M., Paredes A., Lara H.E. (2008). Neonatal Exposure to Estradiol Valerate Programs Ovarian Sympathetic Innervation and Follicular Development in the Adult Rat1. Biol. Reprod..

[7] Matalliotakis I., Kourtis A., Koukoura O., Panidis D. (2006). Polycystic Ovary Syndrome: Etiology and Pathogenesis. Arch. Gynecol. Obstet..

[8] Lara H.E., Ferruz J.L., Luza S., Bustamante D.A., Borges Y., Ojeda S.R. (1993). Activation of Ovarian Sympathetic Nerves in Polycystic Ovary Syndrome. Endocrinology.

[9] Han S.Y., McLennan T., Czieselsky K., Herbison A.E. (2015). Selective Optogenetic Activation of Arcuate Kisspeptin Neurons Generates Pulsatile Luteinizing Hormone Secretion. Proc. Natl. Acad. Sci. USA.

[10] Mayerhofer A., Kunz L., Krieger A., Proskocil B., Spindel E., Amsterdam A., Dissen G.A., Ojeda S.R., Wessler I. (2006). FSH Regulates Acetycholine Production by Ovarian Granulosa Cells. Reprod. Biol. Endocrinol..

[11] Urra J., Blohberger J., Tiszavari M., Mayerhofer A., Lara H.E. (2016). In Vivo Blockade of Acetylcholinesterase Increases Intraovarian Acetylcholine and Enhances Follicular Development and Fertility in the Rat. Sci. Rep..

[12] Burden H.W., Lawrence I.E. (1978). Experimental Studies on the Acetylcholinesterase-Positive Nerves in the Ovary of the Rat. Anat. Rec..

[13] Cruz M.E., Chávez R., Domínguez Casalá R. (1986). Ovulation, Follicular Growth and Ovarian Reactivity to Exogenous Gonadotropins in Adult Rats with Unilateral or Bilateral Section of the Vagi Nerves. Rev. Investig. Clín..

[14] Morales-Ledesma L., Betanzos-García R., Domínguez-Casalá R. (2004). Unilateral or Bilateral Vagotomy Performed on Prepubertal Rats at Puberty Onset of Female Rat Deregulates Ovarian Function. Arch. Med. Res..

[15] Morales L., Ricardo B., Bolaños A., Chavira R., Domínguez R. (2007). Ipsilateral Vagotomy to Unilaterally Ovariectomized Pre-Pubertal Rats Modifies Compensatory Ovarian Responses. Reprod. Biol. Endocrinol..

[16] Linares R., Hernández D., Morán C., Chavira R., Cárdenas M., Domínguez R., Morales-Ledesma L. (2013). Unilateral or Bilateral Vagotomy Induces Ovulation in Both Ovaries of Rats with Polycystic Ovarian Syndrome. Reprod. Biol. Endocrinol..

[17] Linares R., Rosas G., Vieyra E., Ramírez D.A., Velázquez D.R., Espinoza J.A., Morán C., Domínguez R., Morales-Ledesma L. (2019). In Adult Rats With Polycystic Ovarian Syndrome, Unilateral or Bilateral Vagotomy Modifies the Noradrenergic Concentration in the Ovaries and the Celiac Superior Mesenteric Ganglia in Different Ways. Front. Physiol..

[18] Mayerhofer A., Fritz S. (2002). Ovarian Acetylcholine and Muscarinic Receptors: Hints of a Novel Intrinsic Ovarian Regulatory System. Microsc. Res. Tech..

[19] Krsmanovic L.Z., Mores N., Navarro C.E., Saeed S.A., Arora K.K., Catt K.J. (1998). Muscarinic Regulation of Intracellular Signaling and Neurosecretion in Gonadotropin-Releasing Hormone Neurons. Endocrinology.

[20] Dominguez R., Riboni L., Zipitria D., Revilla R. (1982). Is There a Cholinergic Circadian Rhythm throughout the Oestrous Cycle Related to Ovulation in the Rat?. J. Endocrinol..

[21] Morales-Ledesma L., Linares R., Rosas G., Morán C., Chavira R., Cárdenas M., Domínguez R. (2010). Unilateral Sectioning of the Superior Ovarian Nerve of Rats with Polycystic Ovarian Syndrome Restores Ovulation in the Innervated Ovary. Reprod. Biol. Endocrinol..

[22] Venegas-Meneses B., Padilla J.F., Juárez C.E., Morán J.L., Morán C., Rosas-Murrieta N.H., Handal A., Domínguez R. (2015). Effects of Ovarian Dopaminergic Receptors on Ovulation. Endocrine.

[23] Morales L., Chavez R., Ayala M., Dominguez R. (1998). Effects of Unilateral or Bilateral Superior Ovarian Nerve Section in Prepubertal Rats on the Ovulatory Response to Gonadotrophin Administration. J. Endocrinol..

[24] Greenwald G.G. (1994). Follicular development and its control. The Physiology of Reproduction.

[25] Lara H.E., Dissen G.A., Leyton V., Paredes A., Fuenzalida H., Fiedler J.L., Ojeda S.R. (2000). An Increased Intraovarian Synthesis of Nerve Growth Factor and Its Low Affinity Receptor Is a Principal Component of Steroid-Induced Polycystic Ovary in the Rat. Endocrinology.

[26] Dominguez R., Morales-Ledesma L., Cruz M.E. (2003). Ovarian Asymmetry. Annu. Rev. Biomed. Sci..

[27] Venegas B., De León Gordillo L.Y., Rosas G., Espinoza J.A., Morán C., Domínguez R., Morales-Ledesma L. (2019). In Rats with Estradiol Valerate-Induced Polycystic Ovary Syndrome, the Acute Blockade of Ovarian β-Adrenoreceptors Improve Ovulation. Reprod. Biol. Endocrinol. RBE.

[28] Brawer J.R., Munoz M., Farookhi R. (1986). Development of the Polycystic Ovarian Condition (PCO) in the Estradiol Valerate-Treated Rat. Biol. Reprod..

[29] Burden H.W., Leonard M., Smith C.P., Lawrence I.E. (1983). The Sensory Innervation of the Ovary: A Horseradish Peroxidase Study in the Rat. Anat. Rec..

[30] Trkulja V., Lackovic Z. (2001). Vagal Influence on Compensatory Ovarian Growth Is Important Only Briefly after Hemicastration. Exp. Biol. Med..

[31] Stillman M.J., Shukitt-Hale B., Kong R.M., Levy A., Lieberman H.R. (1993). Elevation of Hippocampal Extracellular Acetylcholine Levels by Methoctramine. Brain Res. Bull..

[32] Toide K., Arima T. (1989). Effects of Cholinergic Drugs on Extracellular Levels of Acetylcholine and Choline in Rat Cortex, Hippocampus and Striatum Studied by Brain Dialysis. Eur. J. Pharmacol..

[33] Damsma G., Westerink B.H.C., Vries J.B., Berg C.J., Horn A.S. (1987). Measurement of Acetylcholine Release in Freely Moving Rats by Means of Automated Intracerebral Dialysis. J. Neurochem..

[34] Barco A.I., Flores A., Chavira R., Damián-Matsumura P., Domínguez R., Cruz M.E. (2003). Asymmetric Effects of Acute Hemiovariectomy on Steroid Hormone Secretion by the In Situ Ovary. Endocrine.

[35] Burden H.W., Lawrence I.E. (1977). The Effect of Denervation on Compensatory Ovarian Hypertrophy. Neuroendocrinology.

[36] Klein C.M., Burden H.W. (1988). Anatomical Localization of Afferent and Postganglionic Sympathetic Neurons Innervating the Rat Ovary. Neurosci. Lett..

[37] Fritz S., Grünert R., Stocco D.M., Hales D.B., Mayerhofer A. (2001). StAR Protein Is Increased by Muscarinic Receptor Activation in Human Luteinized Granulosa Cells. Mol. Cell. Endocrinol..

[38] Mayerhofer A., Dimitrijevic N., Kunz L. (2003). The Expression and Biological Role of the Non-Neuronal Cholinergic System in the Ovary. Life Sci..

[39] Fritz S., Föhr K.J., Boddien S., Berg U., Brucker C., Mayerhofer A. (1999). Functional and Molecular Characterization of a Muscarinic Receptor Type and Evidence for Expression of Choline-Acetyltransferase and Vesicular Acetylcholine Transporter in Human Granulosa-Luteal Cells1. J. Clin. Endocrinol. Metab..

[40] Kunz L., Thalhammer A., Berg F.D., Berg U., Duffy D.M., Stouffer R.L., Dissen G.A., Ojeda S.R., Mayerhofer A. (2002). Ca^2+^-Activated, Large Conductance K^+^ Channel in the Ovary: Identification, Characterization, and Functional Involvement in Steroidogenesis. J. Clin. Endocrinol. Metab..

[41] Mayerhofer A., Kunz L. (2005). A Non-Neuronal Cholinergic System of the Ovarian Follicle. Ann. Anat. Anat. Anz..

[42] Flores A., Meléndez G., Palafox M.T., Rodríguez J.O., Barco A.I., Chavira R., Domínguez R., Cruz M.E. (2005). The Participation of the Cholinergic System in Regulating Progesterone Secretion Through the Ovarian–Adrenal Crosstalk Varies Along the Estrous Cycle. Endocrine.

[43] Flores A., Rodríguez J.O., Palafox M.T., Meléndez G., Barco A.I., Chavira R., Esther Cruz M., Domínguez R. (2006). The Acute Asymmetric Effects of Hemiovariectomy on Testosterone Secretion Vary along the Estrous Cycle. The Participation of the Cholinergic System. Reprod. Biol. Endocrinol..

[44] Dominguez R., Cruz-Morales S.-E. (2011). The Ovarian Innervation Participates in the Regulation of Ovarian Functions. Endocrinol. Metab. Syndr..

[45] Michal P., Lysíková M., Tuček S. (2001). Dual Effects of Muscarinic M_2_ Acetylcholine Receptors on the Synthesis of Cyclic AMP in CHO Cells: Dependence on Time, Receptor Density and Receptor Agonists: Muscarinic M_2_ Receptors on Cyclic AMP Synthesis. Br. J. Pharmacol..

[46] Bódis J., Tinneberg H.R., Papenfuß F., Török A., Cledon P., Hanf V., Schwarz H. (1993). Cholinergic Stimulation of Progesterone and Estradiol Secretion by Human Granulosa Cells Cultured in Serum-Free Medium. Gynecol. Endocrinol..

[47] Kornya L., Bódis J., Koppán M., Tinneberg H.R., Török A. (2001). Modulatory Effect of Acetylcholine on Gonadotropin-Stimulated Human Granulosa Cell Steroid Secretion. Gynecol. Obstet. Investig..

[48] Luck M.R. (1990). Cholinergic Stimulation, through Muscarinic Receptors, of Oxytocin and Progesterone Secretion from Bovine Granulosa Cells Undergoing Spontaneous Luteinization in Serum-Free Culture. Endocrinology.

[49] Cruz M.E., Flores A., Alvarado B.E., Hernández C.G., Zárate A., Chavira R., Cárdenas M., Arrieta-Cruz I., Gutiérrez-Juárez R. (2015). Ovulation Requires the Activation on Proestrus of M1 Muscarinic Receptors in the Left Ovary. Endocrine.

[50] Morales L., Chávez R., Domínguez R. (1993). Participation of the Superior Ovarian Nerve in the Regulation of Ovulation in the Prepubertal Rat: Differential Effects of Uni-Lateral and Bilateral Section of the Nerve. Med. Sci. Res..

[51] Riquelme R., Ruz F., Mayerhofer A., Lara H.E. (2020). Huperzine-A Administration Recovers Rat Ovary Function after Sympathetic Stress. J. Neuroendocrinol..

